# Salutogenomics: embracing the full spectrum of human health

**DOI:** 10.1016/j.tig.2025.12.004

**Published:** 2026-01-22

**Authors:** Noah Collins, Grace Heyborne, Agustín Fuentes, Justin Lund, Melissa Ilardo

**Affiliations:** 1 Department of Anthropology, Princeton University, Princeton, NJ, USA; 2 Department of Biomedical Informatics, University of Utah, Salt Lake City, UT, USA; 3 Department of Anatomy and Cell Biology, Oklahoma State University Center for Health Sciences, Tulsa, OK, USA; 4 Division of Anthropology, California State University, Fullerton, Fullerton, CA, USA; 5 Department of Human Genetics, University of Utah, Salt Lake City, UT, USA; 6 These authors equally contributed to this work.

## Abstract

Modern biomedical genomics has principally centered on disease, leveraging genomic insights to identify disease-associated genotypes. While valuable, this approach is overly reliant on a positivist, disease-centric perspective that often goes hand-in-hand with patterns like deficit framing, racialized medical stereotyping, and genetic determinism. These practices and their underlying beliefs are detrimental for patients, who experience worse health outcomes as a result, and for participant communities, who endure associated stigmas. This commentary seeks to examine the consequences of this narrow lens and to describe the benefits of an alternative approach: salutogenomics, which highlights the full spectrum of human health. Additionally, we explore how adopting diverse knowledge production paradigms could refashion Western genomic methodologies.

## Salutogenomics—challenging paradigms

The hunt for disease-causing genetic variants has long been guided by a positivist framework, rooted in the assumption that a singular, objective truth about disease causality awaits discovery by scientists. This assumption, however, proves insufficient in light of individual- and population-level variations. Furthermore, this approach has become entangled in reductive dichotomies that frame study participants as ‘us’ versus ‘them’, placing the burden of causality disproportionately on participant communities and their bodies. Such framings risk reinforcing marginalization and mischaracterization of entire populations.

This persistence can be attributed, in part, to computational and logistical convenience: it is far easier to detect the disruption of biological function than the protection against it. This bias is exacerbated by the structure of electronic health record data, which remains the most abundant source for biomedical genomic research but inherently lacks information on the absence of disease. Health exists on a spectrum, and within that spectrum lies a vast array of genetic variation that promotes the very opposite of disease and disruption: adaptation and resilience. It is imperative that the biomedical field considers its own theoretical framings and positionality to address the consequences of its methodologies and the well-being of its participants.

To that end, we offer a complementary paradigm: salutogenomics. Derived from the concept of salutogenesis, which emphasizes health and resilience over pathology, salutogenomics represents a resilience-oriented framework for genomic research. Salutogenomic research seeks to understand how health is preserved among individuals who seem protected from disease or otherwise resilient. This approach often focuses on the interests and goals of communities, especially as they are more willing to engage in research that does not stigmatize them. Indigenous community–based participatory research models frequently illuminate genomic factors that foster resilience and/or protect against disease, as opposed to the factors that cause disease. Salutogenomics builds on this by centering a community’s particular genomic structures as strengths to be celebrated rather than weaknesses to be expunged. This effort enhances the understanding of the resilience of participant populations and follows a growing trend aimed toward advancing Western medical science in meaningful ways by reflecting on problematic positionalities and perspectives that contribute to community stigmatization and mischaracterization while failing to highlight core human variation and resilience. This salutogenomic framing emerges as part of multiple initiatives to increase ethical engagement with participant communities, which has, in turn, built trust and yielded significant scientific insight [1–5].

As authors, we represent an interdisciplinary blend of epistemologies, anthropological and biomedical frameworks, and genomic expertise. While all authors are from the USA, we regularly engage in international research and are concerned with the well-being of global and domestic communities. This manuscript reflects a shared commitment to advancing interdisciplinary collaboration and promoting diversity of perspectives in genomic science to augment the impact of our respective disciplines and health outcomes. Our intention is to highlight the potential harms of unexamined positionalities and an over-reliance on positivist frameworks in medical genomics research, while underscoring the value of embracing diverse perspectives and the full spectrum of human experience, knowledge systems, and health.

To do this, we address the ways in which the current disease-centric approach can prove harmful, namely through deficit framing, medical stereotypes, and genetic determinism. We frame the consequences of this approach in the context of genomic health research, emphasizing its exacerbated effect on Indigenous communities. Subsequently, we discuss alternative approaches that push paradigm boundaries, like those born from the realm of Native American and Indigenous health research, where alternative ways of knowing have benefited scientific inquiry. We then characterize a salutogenomic model for approaching genomics research that accounts for previously neglected aspects of the human health spectrum and provide examples of health- and resilience-oriented genomic research in order to demonstrate its value. Our goal is to highlight that diverse methodologies will continue to enable effective, genetically anchored biomedical insights that improve health and inspire collaborative input.

## Disease perspective—how it can harm

A disease-centric perspective is the primary lens through which biomedical genomic research approaches human health. Much of what we know about human health is defined by the identification of pathogenic biological variants. While such observations have value, evidenced by modern advancements in the treatment of disease, this approach has also had harmful consequences.

One such consequence is born from the very perspective that defines this disease-centric approach: deficit framing. Health researchers often define and investigate genetic variation through the presence of disease [6] (i.e., the deficit). Exclusively highlighting the ways in which participants lack wellness can simultaneously associate entire communities and/or ancestries with a particular ‘deficit’ while shifting attention away from important determinants of health related to the disease. In the deficit model, such groups are often labeled as ‘disease cohorts’ (from cohort study designs), an evocative framing that, in itself, can shame participants via their assumed biological connection to the disease. This contributes to the narrative that researchers can ‘fix what is wrong’ in these communities through positivist, targeted genetic investigation.

Association is a powerful force within biomedical research and medicine, but it is an equally strong social force. Once the frame of ‘disease cohort’ has been applied to a community, it is nearly impossible to remove the association of the disease with the group. For example, ‘thrifty gene’ and ‘warrior gene’ narratives dominate racialized perceptions of entire populations as being ‘doomed’ to the effects of genetic variants [7]. In the case of thrifty gene narratives, communities and individuals from those populations can lose motivation to have a healthy diet or to exercise because they believe that their fitness and general health are overwhelmingly predetermined by genes, the outcome unchanging regardless of actions taken [8]. This kind of marginalization can result in communities onboarding harmful and pathological narratives about themselves.

Negative associations and an overburden of deficit-framed research can also discourage communities from engaging in biomedical and genomic research, leading to further stigmatization and even research fatigue [9]. Efforts to converse with communities, to understand their perspectives, or to prioritize their immediate health concerns are becoming more common [10,11] but need to become the expectation rather than the exception. When already marginalized communities do not feel understood or valued by researchers, they are less likely to participate in research [12]. Additionally, when populations become framed as the default population in which to study a common phenotype or genotype, it can lead to an influx of research dedicated to that topic, such as the Pine Ridge Reservation concerning alcohol, poverty, and suicide [13]. As a result, the disparities that already affect these groups widen. The gap in genetic data diversity [14] both disproportionately harms marginalized communities [15] and cannot be closed without their active participation.

Biases of clinicians are often based on stereotypes that, in part, arise from deficit-framed research and can easily lead to the practice of race-based medicine [16]. Studies show that medical practitioners are likely to perceive race-based stereotypes as factual, despite also understanding the inherent harm of these stereotypes [17]. Race is notoriously misused in medical and other biological research as an objective biological variable, when it inherently relies on nonbiological social constructions that vary across both geography and culture. Correlating variation in health with race relies on the convention that racial groups are genetically and biologically distinct from one another, which has been systematically and repeatedly disproven for decades. Quite simply, humans cannot be genetically divided into biological subcategories that correspond with race [18]. The continuous repetition of this misconception in medical schools reinforces the false validity of race-based medicine and increases its use in clinical practice [19]. Here, racial bias is so intricately woven into the medical system that it is difficult to educate future clinicians without affirming it.

Beyond clinician bias, even statistical tools and medical devices contribute to the creation of inequities and the perpetuation of racial misconceptions. For example, the use of polygenic risk scores (PRSs), scores based on the culmination of variants that are collectively associated with disease risk, offers many clinical benefits for predicting individual risk and diagnosing disease [20]; however, PRSs are not immune to bias. In fact, there is uncertainty as to the predictive power of PRS estimates and their accuracy across different ancestral groups [21], likely due to the European genome bias in genome-wide association studies [14]. Additionally, medical devices such as pulse oximeters, which measure oxygen saturation via light absorption, are notoriously biased toward different groups. Due to differential light absorption patterns, readings for patients with darker skin pigmentation are predicted to be likely 2.5× less accurate [22]. Despite the clear documentation of this manufacturing bias and its harm, the demand for better calibration algorithms has not been met [23]. In these ways, the unreliability of quantitative and technological outputs can reaffirm pre-existing racial beliefs about both biology and the objectivity of biological measurements. Such measurements are then susceptible to reinforcing clinical biases against marginalized groups, which can perpetuate a belief in genetic determinism.

Genetic determinism, the belief that genes have complete or predominant causal power over the traits that we observe, is an oversimplification of genetic causation [24] that can compound with the harms of stereotyping. Such determinism is often sustained by a reliance on Mendelian models that frame disease phenotypes as having monogenic, single-variant origins. Many diseases and phenotypes are not monogenic; rather, they are complex traits shaped by a combination of multiple genetic, social, and environmental factors. The assumption that monogenic variants are causal for all complex phenotypes (and associated stereotypes) validates inaccurate associations and reinforces bias, both of which ultimately support race-based and deficit-framed medicine. Such approaches create a cyclical relationship where one (genetic determinism) feeds into another (stereotyping and race-based medicine), which is then used as evidence to justify itself.

Deficit framing and positivist approaches, which inform medicine through the characterization of disease, ultimately seek to identify genetic variation as a means to improve human health. This benevolent intention does not, however, grant these orientations immunity from contributing to stigmatization, marginalization, and the subsequent harm of participant communities. In addressing this reality, biomedical genomics has the power to contribute to a significant change: a change that reduces disparity within research and medicine alike while prioritizing the just treatment of participant communities.

## Indigenous research and associated methodologies—contributing to salutogenomics

Historically, research has been conducted on Indigenous communities rather than in collaboration with or led by them. In the past and contemporarily, genomics research has harmed Indigenous participants who trusted researchers [25,26]. Today, many frameworks are being positioned as the ‘gold standard’ for engagement with participant communities [1,2,4,27]. While they share many central philosophies, such as the concepts of decolonization, relevance, respect, and reciprocity, there are no frameworks that can be copied from one cultural context into another. Research that explores relevant topics in respectful ways with equal benefits to participants remains the only appropriate way to collaborate with participant communities. Populations around the world are uninterested in being deficit-framed, such as after having been studied for decades on end and framed as ‘the alcoholic community’ or ‘the diabetes community’, thus becoming the default for many pathological inquiries. Emerging published philosophies encourage resilience frameworks as appropriately situated to create future research equity and more robust health narratives. Rather than centralizing epidemiological deficits, Indigenous research methodologies offer a shift to contextualized resiliency and beneficial exception.

Aside from advancing decolonization, Indigenous research methodologies are approaches that center community-driven questions and values, are grounded in local and cultural contexts, and emphasize health, well-being, and respect—all of which align with a salutogenomics approach. One central component of Indigenous research methodologies is a challenge to the objectivity of the scientific gaze [28]. Rather than being an objective observer of a ‘truth’, the scientist is always a subjective participant in the creation of situated knowledge. Deficit framing, particularly of non-Western communities, is uniquely built into Western scientific discovery, where objectivity is the gold standard. Breaking away from the positivist paradigm to explore human health from various wellness perspectives aligns with movements that explicitly shift away from Western perspectives and fits more effectively with the current state of knowledge about human genomic systems. Western scientific inquiry can be beneficially shaped and restructured by true collaboration with Indigenous research, and such a dynamic benefits from a salutogenomics perspective.

Codesign, respect for persons, and going beyond field ethical standards build trust and foster equitable science that reflects powerful narratives about research communities. Good or bad, Western methodological practices have a powerful legacy; however, thinking beyond the boundaries of research paradigms is not only important but also ethical and may lead to more research for improved health care outcomes.

## Genetic resilience—what it is and why it matters

Genetic variation underlies the full spectrum of human phenotypes, from health to disease; however, it is deleterious variants that have received the most attention in biomedical genomics, in part because they are easier to identify using Western methodological practices—disease phenotypes are more easily recognizable than phenotypes that protect against disease (resilience phenotypes). Consequently, resilience-focused inquiry sits outside the aforementioned Western paradigm boundary and thus offers us a means to traverse it. Despite being less common than deleterious alleles, protective alleles are known to be present within the human genome [29]. These protective variants enable humans to inhabit some of the harshest climates on Earth, eat a highly variable diet, and overcome a wide variety of physiological stressors [30]. This protective genetic variation is what we refer to as resilience: a genetic component in the human ability to adapt and thrive in the face of physiological pressures.

Resilience has been observed across populations and is associated with numerous biological mechanisms. The increasing acceptance of resilience phenotypes coincides with an important shift happening within genomics: a shift away from racialized, and often eugenics-based, ideas of ‘normal’ and toward a more nuanced perspective in which variation itself is normal. To assume the existence of an objective ‘normal’ limits the scope of inquiry to variation that is ‘abnormal’, effectively reducing the power of our genomic understanding. Perhaps one of the best-documented examples of resilient variation is that of high-altitude adaptations. Such variants are associated with high-altitude populations in the Himalayas, Andes, and Ethiopia, with the specific variants differing between these groups.

The Himalayan populations of Tibetan and Sherpa have been associated with positively selected variants in genes related to the hypoxia-inducible factor (HIF), which affects the oxygen-sensing pathway: *EPAS1* and *EGLN1* [31–34]. *EPAS1* encodes for the protein HIF-1α, and *EGLN1* subsequently encodes for the enzyme PHD2, which targets HIF-2α for degradation in normal oxygen environments. This pathway helps regulate the hypoxia response, and variations in the pathway can help mitigate the harmful effects of hypoxia, such as elevated hemoglobin (polycythemia). These two genes do not exclusively influence hypoxia, though, and thus, multiple other genes have also been associated with high-altitude adaptation in Himalayan populations [35].

There is some overlap between observed variation in Himalayan and Andean populations, like with *EGLN1* and *EPAS* [32,36], but there are many distinct differences. In Andean populations, positively selected variants also include genes related to the nitric oxide pathway and the cardiovascular system: *NOS2*, *NOS2A*, and *EDNRA* [32,37,38], which, respectively, encode for nitric oxide synthase and are involved with vasoconstriction. Ethiopian highland populations, interestingly, differ from the variants observed in both Andeans and Tibetans. They possess two independent genetic variants associated with the HIF pathway in *THRB* and *ARNT2* [39]. The variation in genetic adaptation across these three populations illustrates both the complexity of resilience and the merit of future research on protective variants. Understanding these genes through their adaptive histories can reveal relevant mechanisms and phenotypes to inform therapeutic efforts.

In addition to altitude adaptations, recent research has discovered protective adaptations related to the practice of breath-hold diving in marine hunter-gatherers. The dive response experienced by diving mammals is very similar to the dive response experienced by humans and includes the contraction of the spleen to release oxygenated red blood cells into the circulatory system [40]. A genetic variant in the *PDE10A* gene was associated with increased spleen size in Bajau (a diving population inhabiting Southeast Asia), providing them with a larger reservoir of oxygenated red blood cells [41], which has allowed them to better forage their marine-adjacent environment. In addition, genetic variation has been discovered in the Haenyeo, all-female divers of Korea, which may mitigate the dangers of diving throughout pregnancy [42].

In addition to hypoxia driven by altitude and diving, pathogen exposure is also a driver of resilience. A recent study investigated protection from Chagas disease in Amazonian populations, where the ecological conditions make inhabitants particularly susceptible to the transmission of diseases. The study found a strong selective signal in genes related to pathogen resistance: *PPP3CA*, *DYNC1I1*, and *NOS1AP*. A variant of the *PPP3CA* gene, specifically a gene associated with immune response and *Trypanosoma cruzi*—the pathogen responsible for Chagas, has likely provided Amazonian populations protection against the disease in the past, possibly by reducing the infection rate among carriers [43]. Research of this nature is increasingly valuable, as concerns around future global pandemics, decreased biodiversity, and climate change continue to grow. Novel research in this area is not only in the best interest of the scientific community but also of humanity at large.

Beyond the Amazon, similar investigations have uncovered or proposed protective variants across diverse populations, offering further insights into the genetic basis of resilience. In the Māori and other Pacific populations in New Zealand, a *CREBRF* variant has been associated with type 2 diabetes protection [44]. In the Tsimane, an Indigenous group living in lowland Bolivia, a particularly low rate of dementia has been observed, which may be the result of adaptive protection, though it has not yet been genetically linked [45]. In African Americans, *GRK2* and *GRK5* variants have been associated with decreased mortality due to heart failure or cardiac ischemia [46].

The study of Alzheimer’s disease has also revealed protective variants. Alzheimer’s is a progressive neurodegenerative disorder, but a large number of individuals who display neuropathological indicators for Alzheimer’s are asymptomatic and do not show clinical signs of cognitive impairment. In fact, 70% of cognitively unimpaired adults have some Alzheimer’s indicators, and nearly 30% would meet Alzheimer’s diagnostic criteria [47,48]. This suggests the possibility of protective biological mechanisms against Alzheimer’s. Thus far, over a dozen possible protective variants have been proposed [48]. The best documented variants included an *APOE*ε2 variant [49] and a *PLCG2* variant [50], which have both been associated with reduced Alzheimer’s risk.

These studies illustrate the complexity and the variability of human adaptation and showcase what we already know: humans are resilient. As a feature of human evolution and its concomitant genetic variation, humans have the potential to adapt to a plethora of climates and situations. To ignore this is to ignore the reality of a human health spectrum and the dynamic genetic variation associated with it.

## Conclusion

In this commentary, we offer that the current epistemological framework through which Western medical science understands and investigates human health is overly reliant on deficit framing and a positivist perspective. We suggest that there are important consequences of the Western knowledge production paradigm, including the reinforcement of harmful stereotypes, the perpetuation of race-based medicine, and the affirmation of genetic determinism, all of which harm marginalized groups and introduce barriers to health equity and greater understanding of human systems of resilience. We acknowledge that there is room for continued study of deleterious genetic variants; however, we suggest that the current approach is too limited, pathologizes variation, contributes to problematic practices and beliefs, and fails to utilize diverse perspectives and ways of knowing. It is these qualities that we believe the field of biomedical genomics should move away from.

We offer a solution: a salutogenomic approach, one that centers resilience rather than pathology, prioritizes the identification of protective genetic variants, and embraces diverse epistemologies to more ethically and accurately reflect the full spectrum of human health and variation (for more on future research directions, see the Outstanding questions). This approach challenges, without invalidating, the pervasive philosophy that has dominated biomedical genomics. We suggest that the addition of a resilience perspective and insights from research by Indigenous scholars and communities can help mitigate health disparities in several important ways. First, it brings attention to the full spectrum of human health, which can aid in dismantling the segregation of disease cohorts and the harmful stereotypes forced upon participant communities. Second, it encourages the use of novel and progressive research orientations that approach human health from unique perspectives and cater to community needs. Finally, it increases appreciation for the dynamic genetic variation that underlies human health—variation with potential protective qualities that may be implicated in both creating human health and the treatment of disease. A shift toward incorporating diverse approaches, including a salutogenomic resilience-oriented approach to health research, stands to benefit researchers, clinicians, and participant communities alike. We have everything to gain from the addition of a salutogenomic perspective if we choose to challenge traditionally practiced Western epistemologies and embrace the nuances of human health.

## Highlights

Research involving marginalized populations often centers on disease, which can pathologize these communities and reinforce harmful stigmas.

The persistence of population stereotyping presents a significant challenge for health practitioners and researchers alike, who must contend with racialized medical assumptions and reductionist beliefs such as genetic determinism.

The emergence of resilience phenotypes across multiple human health domains has demonstrated the validity of genomic resilience research and its associated methodologies.

Despite growing evidence for protective variants, resilience remains an underexplored area of study.

## Outstanding questions

Current genomic methodologies are tailored to identify pathogenic variants and diseases. What methods remain to be developed to identify resilience phenotypes?

Health records almost exclusively document the absence of health, not the presence of it. How can computational gaps be filled to improve the feasibility of this approach for large-scale research?

The European genome bias cannot be addressed without the active involvement of traditionally marginalized communities, who must feel safe to participate in and empowered to conduct genomic research. How can researchers and research institutions alike encourage data diversity?

The mechanisms and population specificity of protective variants require further exploration. How do protective genetic variants influence health outcomes across diverse populations?

While the existence of protective variants alone is significant, the application of these variants to disease treatment and prevention remains understudied. How can resilience data and protective genetic variants aid in the treatment and prevention of disease?

Reframing human health research has the potential to reduce the pathologization of marginalized communities and promote equity-driven inquiry. Following the implementation of this perspective, we must ask: does the inclusion of a resilience framework in health research improve health equity?
